# Integrated Cognitive and Neuromotor Rehabilitation in Multiple Sclerosis: A Pragmatic Study

**DOI:** 10.3389/fnbeh.2018.00196

**Published:** 2018-09-05

**Authors:** Anna M. Barbarulo, Giacomo Lus, Elisabetta Signoriello, Luigi Trojano, Dario Grossi, Mariateresa Esposito, Teresa Costabile, Roberta Lanzillo, Francesco Saccà, Vincenzo Brescia Morra, Giovannina Conchiglia

**Affiliations:** ^1^Multiple Sclerosis Center, II Division of Neurology, Department of Surgical Medical Science, Neurological, Metabolic and Aging, University of Campania Luigi Vanvitelli, Naples, Italy; ^2^Interuniversity Center for Research in Neurosciences, University of Campania Luigi Vanvitelli, Naples, Italy; ^3^Department of Psychology, University of Campania Luigi Vanvitelli, Caserta, Italy; ^4^Department of Neurosciences, Reproductive and Odontostomatological Sciences, University of Naples Federico II, Naples, Italy; ^5^Foundation Villa Camaldoli, Rehabilitation Clinic Alma Mater S.p.A., Naples, Italy

**Keywords:** multiple sclerosis, cognitive rehabilitation, neuromotor rehabilitation, brain plasticity, cognitive motor interference

## Abstract

**Background:** Few studies examined the effects of combined motor and cognitive rehabilitation in patients with multiple sclerosis (MS). The present prospective, multicenter, observational study aimed to determine the efficacy of an integrated cognitive and neuromotor rehabilitation program versus a traditional neuromotor training on walking, balance, cognition and emotional functioning in MS patients.

**Methods:** Sixty three MS patients were selected and assigned either to the Integrated Treatment Group (ITG; *n* = 32), receiving neuropsychological treatment (performed by ERICA software and paper–pencil tasks) complemented by conventional neuromotor rehabilitation, or to the Motor Treatment Group (*n* = 31) receiving neuromotor rehabilitation only. The intervention included two 60-min sessions per week for 24 weeks. At baseline and at end of the training all patients underwent a wide-range neuropsychological, psychological/emotional, and motor assessment.

**Results:** At baseline the two groups did not differ for demographic, neuropsychological, psychological/emotional, and motor features significantly. After rehabilitation, only ITG group significantly (*p-corrected* for False Discovery Rate) improved on test tapping spatial memory, attention and cognitive flexibility, as well as on scales assessing depression and motor performance (balance and gait). A regression analysis showed that neuropsychological and motor improvement was not related to improvements in fatigue and depression.

**Conclusion:** The present study demonstrated positive effects in emotional, motor, and cognitive aspects in MS patients who received an integrated cognitive and neuromotor training. Overall, results are supportive of interventions combining motor and cognitive training for MS.

## Introduction

Multiple sclerosis (MS) is a chronic, autoimmune, and neurodegenerative disorder characterized by inflammation and progressive demyelination of the central nervous system (Hemmer et al., 2006). Deficits in gait and balance, as well as cognitive dysfunctions and reduced cognitive processing speed are among the most common symptoms (Mercan et al., 2016). These deficits are often simultaneously present in patients with MS and this co-occurence could affect the rehabilitation outcomes (Felippe et al., 2018). In the present study we investigated whether a rehabilitation program tackling both cognitive and neuromotor impairments could improve walking, balance, cognition, and emotional functioning in MS.

Approximately 85% of patients with MS experience clinically significant walking disturbances, which may be present since early stages of the disease and in patients with mild disability (Motl and Learmonth, 2014). Balance dysfunction can be present even in the absence of clinical disability (Martin et al., 2006). Impaired walking is increasingly being recognized among factors affecting quality of life, since mobility was given the highest priority by 65% of patients with MS (Paltamaa et al., 2007). Gait deviations predict patient dependence, with slower speed, shorter stride length, and decreased distance walked, largely contributing to patients’ perception of their own ability to perform daily activities (Paltamaa et al., 2007). For these reasons, walking and balance should be regularly assessed (Halabchi et al., 2017).

Prevalence of cognitive deficits ranges 43 to 70% at both early and late stages of MS (Sumowski et al., 2018). Patients’ neuropsychological profile includes deficits in attention, working memory, processing speed, verbal and spatial memory, verbal fluency and executive functioning (Benedict et al., 2017) particularly in progressive MS, but also in Relapsing Remitting MS (Amato et al., 2006b). Cognitive impairment can be as detrimental as motor disturbances on quality of life, functional adaptation, and health perception in MS (Saccà et al., 2016; Kratz et al., 2017).

Motor and cognitive impairments have been often examined independently, but they can interact with each other, as shown by studies on simultaneous motor and cognitive performance (Leone et al., 2015; Learmonth et al., 2017) and by patients’ difficulties in performing daily activities simultaneously, e.g., conversing while walking outdoors. Indeed, walking requires high-order information processing (Sandroff et al., 2014). Patients with impaired attention, working memory, or reasoning abilities may be prone to errors in executing motor-based tasks, and be at high risk for accidents (Kalron, 2014). Benedict et al. (2011) showed that processing speed and executive functioning were significant predictors of lower and upper motor function in healthy individuals and MS patients, but these correlations were more robust in the MS group, where cognitive tests predicted motor function after controlling for disease duration and physical disability. Butchard-MacDonald et al. (2017) reported that relapsing-remitting MS causes difficulties in dual task conditions, with an impact on balance and risk of falls, and also identified a relationship between anxiety/depression and decreased efficiency in dual task. Thus, comprehending the relationships between cognition and functional motor outcomes has strong implications for development of risk assessment procedures and rehabilitation treatments in MS (Khan and Amatya, 2017).

Rehabilitation is recognized to be important in ameliorating motor and cognitive functions, reducing disease burden, and improving quality of life in patients with MS (Prosperini et al., 2015c; Goverover et al., 2017). Classically, motor training has been considered as an approach to improve walking function, and cognitive rehabilitation as an approach to improve cognitive function, separately (Prosperini et al., 2015c). However, combined rehabilitation interventions might simultaneously improve walking and cognition, perhaps due to cognitive motor coupling and/or cross-modality transfer effects (Prosperini et al., 2015a; Motl et al., 2016). Based on evidence from other neurological populations (Wajda et al., 2017) a combined neuromotor and cognitive rehabilitation training can serve as a potential approach to improve both walking and cognitive functioning in MS. There is some preliminary evidence for cross-modality transfer effects regarding exercise training in MS (Motl and McAuley, 2014; Sandroff et al., 2014, 2017). For instance, studies on aerobic exercise training and on physical activity interventions reported co-occurring improvements in cognitive and motor outcomes (Mofateh et al., 2017). In particular, the cognitive training combined with the aerobic exercises proved effective to improve cognitive performance (Jimenez-Morales et al., 2017). For example, Sangelaji et al. (2015) demonstrated that physical interventions could improve cognitive abilities (long-term storage and permanent long-term retrieval of information) in MS patients. Briken et al. (2014) showed beneficial effects of exercise on physical measures (aerobic fitness and walking ability), as well as on neuropsychiatric symptoms (cognitive impairment, depressive symptoms, and fatigue) in a sample of patients with progressive MS and moderate disability.

Overall, there is promising evidence for aerobic and resistance exercise improving walking performance, and preliminary evidence supporting aerobic exercise for improving cognitive performance in MS (Dalgas et al., 2008). However, cognitive therapies are not yet being used for potentially improving motor disability, although cognitive-motor interactions powerfully activate motor performance (Sosnoff et al., 2017), possibly reducing total time needed for treatment, and cost of care. For instance, Sosnoff et al. (2017) reported that a dual task training program, incorporating cognitive tasks in balance and walking training, improved gait speed and visuospatial memory in a sample of MS patients compared to a single task training program focused on balance and walking function only.

The current investigation examined the feasibility of an integrated cognitive and neuromotor rehabilitative (ICNR) program on walking, balance, cognition, and emotional functioning in a sample of MS patients. We specifically examined the effects of ICNR in terms of improvements of both cognitive and motor performance. We also used specific and validated tests for assessing depression and fatigue, which are very frequent symptoms of MS (Amato et al., 2001; Jones et al., 2018).

## Materials and Methods

### Study Design and Setting

This was a prospective, multicenter, observational study.

We invited all consecutive MS patients attending their regular clinical follow-up visits in two Italian Centers (Second Division of Neurology, Department of Medical, Surgical, Neurologic, Metabolic and Aging Sciences, University of Campania Luigi Vanvitelli, Naples and Department of Neurosciences, Reproductive and Odontostomatological Sciences, University of Naples Federico II) to participate in the study. Recruitment and enrollment started from June 2017.

Inclusion criteria for the study were: (a) diagnosis of MS based on standard International criteria (Polman et al., 2011); (b) age 20–65 years; (c) impairment in at least one neuropsychological test (see below); (d) adequate vision and hearing.

Exclusion criteria were: (a) current or past neurological disorder other than MS; (b) major psychiatric illness; (c) history of learning disability, severe head trauma, alcohol or drug abuse; (d) illiteracy or non-native Italian-speaking individuals; (e) relapse and/or corticosteroid use within 4 weeks before study entry; (f) change of symptomatic treatments in the last 6 months.

**FIGURE 1:**
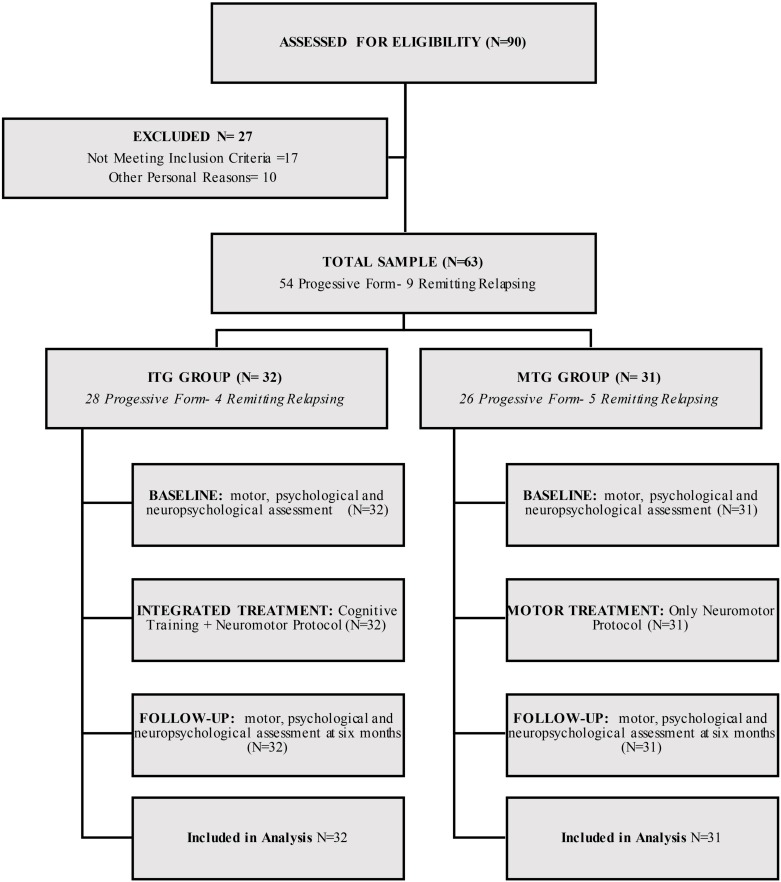
Flowchart illustrating patient selection and study design.

On the basis of selection crieria, from an initial cohort of 90 MS patients we selected 63 individuals (**Figure 1**). Participants were excluded if they did not meet inclusion criteria or could not attend rehabilitation center on a regular basis for logistic reasons (e.g., public transportation or job-related issues); logistic considerations also conditioned assignment of patients to either treatment group.

All participants provided their written informed consent; the study was performed in accordance with the ethical standards laid down in the 1964 Declaration of Helsinki and was approved by the local ethics committee. The study was approved on 11/05/2017 Number of protocol 310.

The Integrated Treatment Group (ITG) received cognitive and motor rehabilitation for 6 months. The control group performed the neuromotor rehabilitation protocol [Motor Treatment Group (MTG)] only. Rehabilitation was carried out at Foundation Villa Camaldoli, Rehabilitation Clinic Alma Mater S.p.A.

All patients underwent a motor, psychiatric and neuropsychological assessment at baseline and at the end of treatment (i.e., 6 months after the baseline evaluation). Post-treatment assessment was performed within the first week after completing the intervention at referral clinics.

The study was double-blinded: the patients were not aware about the rationale and the specific predictions of the study; similarly, the psychologist who carried out the neuropsychological baseline [T0] and post-treatment [T1] assessments, as well as the primary researcher and data entry assistants were blinded to the group membership of the patients.

### Clinical and Neuropsychological Assessment

We collected demographic data (age, sex, level of education) and information about medical history, disease duration, level of disability on the Expanded Disability Status Scale (EDSS, Kurtzke, 1983) and current pharmacological treatment.

The patients underwent 17 neuropsychological tests tapping several cognitive domains (memory, attention, language, executive functions, and reasoning). Each cognitive domain was examined by means of more than one cognitive test. The neuropsychological evaluation included the Italian version of the Rao’s Brief Repeatable Battery (BRB, Amato et al., 2006a). Alternative forms were used in order to reduce test–retest effects and learning effects (Goretti et al., 2014). All scores were corrected by age and education, according to published norms, and performance was considered impaired if its corrected score fell below 1.5 SD.

We also administered further neuropsychological standardized measures assessing: short-term memory (Forward and Backward verbal span, Monaco et al., 2013; Spatial Span, Orsini et al., 1987), selective attention, cognitive flexibility and processing speed (Stroop test, Caffarra et al., 2002), global executive functioning [Frontal Assessment Battery (FAB), Appollonio et al., 2005], non-verbal abstract reasoning [Raven’s Coloured Matrices (RCPMs), Carlesimo et al., 1996], verbal fluency and cognitive flexibility [Phonological Verbal Fluency task (PVF), Carlesimo et al., 1996]. All scores were corrected for age and education, and converted to equivalent scores allowing comparison with the distribution of Italian normative values: an equivalent score of 0 is below the normal range, 1 within normal limits, and 2–4 is within the normal range.

### Psychological Assessment

All patients also underwent a behavioral assessment for anxiety [State-Trait Anxiety Inventory (STAI-Y), including two scales for evaluating temporary condition of “state anxiety,” *A-State*, and long-standing condition of “trait anxiety,” *A-Trait*, Spielberger et al., 1983; Pedrabissi and Santinello, 1998], for depressive symptoms [Beck Depression Inventory-Revised (BDI-II), including the cognitive and the somatic subscales, Beck et al., 1961; Ghisi et al., 2006], and fatigue [Fatigue Severity Scale (FSS), Krupp et al., 1989].

### Motor Function Assessment

For assessing motor/functional status we used the Tinetti Gait and Balance Instrument (to determine fall risk in neurological patients, Tinetti, 1986), and Barthel Index Modified (BIM, assessing independence in activities of daily living, Shah et al., 1989).

### Treatments – Cognitive Rehabilitation

Cognitive Rehabilitation was performed by means of the ERICA Software (Inzaghi, 2013; an Italian computer-assisted tool including exercises in five areas: Attention, Spatial Cognition, Memory, Verbal and Non-verbal executive functions), and of paper and pencil tasks (different from those used in the neuropsychological assessment to prevent the effect of practice). Cognitive Rehabilitation included a training for executive functions, comprising dual task exercises, plus additional exercises tailored on single patient’s neuropsychological impairments. If a patient was impaired in more than one domain, trainings for impaired domains were balanced within sessions. Exercise complexity was adapted to severity of single patient’s impairment in the selected domain, to be challenging in each session (Mattioli et al., 2016). The basic “functionalistic” approach consisted in activating defective cognitive processes, as foreseen in neuropsychological rehabilitation for patients with focal lesions (Mazzucchi, 2012).

### Treatments – Neuromotor Rehabilitation

Conventional Neuromotor treatment aimed at recovery of trunk control, attainment of upright position, load transfer from one buttock to the other, re-education of the step cycle and balance control in static and in dynamic conditions (Lipp and Tomassini, 2015). Problems with balance and coordination are common in MS so the treatment included exercises for increasing stability during gait, preventing falls, and enhancing posture control.

Both groups received an individualized progressive regimen of balance and gait exercises. The difficulty of the exercises was matched to the abilities of the individual participants. Furthermore, motor exercises also aimed at rehabiliting pelvic floor dysfunctions, which limit everyday activities and affect social relationships (Ferreira et al., 2016).

Integrated Treatment Group group received an integrated training consisting in two 60-min sessions per week for 24 consecutive weeks: cognitive training (30 min per session) was complemented with 30 min of a neuromotor rehabilitation protocol. The MTG group received two 60-min sessions per week for 24 consecutive weeks, but the training consisted in 60 min neuromotor rehabilitation.

### Statistical Analysis

All analyses were performed with SPSS, version 20.

The normality assumption for the data was first examined by Kolmogorov–Smirnov test. Descriptive statistics are espressed as median and/or means ± standard deviation for all variables.

Due to the nature of the variables and the sample size, non-parametric tests were performed.

Intergroup differences on baseline characteristics were evaluated by Mann–Whitney’s *U* statistic test for quantitative variables. Chi-squared test was applied to qualitative data.

Intragroup differences (*Repeated measures within group*) were analyzed by Wilconxon signed ranks test (over two-time points) to search for improvements after training. Change values for the outcome measures were calculated by subtracting the baseline data (T0) from the post-intervention data T1 (*Delta = T1–T0*). To analyze intergroup improvement, the “change values (Delta)” were compared using Mann–Whitney’s *U* statistic test with group as a factor.

In line with Mattioli et al. (2010), a linear regression was conducted with the change (Delta) in number of pathological tests as the dependent variable and the following variables as predictors: group (treatment), age, EDSS and Delta scores for depression (BDI-II overall scale), fatigue (FSS) and motor functioning (Tinetti overall Scale), in order to control for changes in depression, fatigue and motor functioning affecting cognitive changes.

Another linear regression analysis was used to determine predictors of motor change (dependent variable: Delta for Tinetti Scale). Variables entered into the model included group (factor), age, EDSS, as well as Delta scores for BDI-II, fatigue (FSS) and number of pathological cognitive tests, in order to control for changes in depression, fatigue, and cognition functioning affecting motor changes.

Statistical significance was set as *p* < 0.05. For reducing risk of Type-I error in statistical analyses on Delta scores for neuropsychological, motor and psychological variables, we took into account *p*-values corrected for familywise error, according to the False Discovery Rate procedure (Benjamini and Hochberg, 1995).

## Results

### Characteristics of the Study Sample

#### Baseline

The sample consisted of 63 patients with MS, most of whom affected by progressive MS; all patients were treated with disease-modifying drugs (DMDs). Thirty-two patients were included in the ITG and 31 in the MTG. The two groups did not differ for demographic and clinical characteristics (**Table 1**).

**Table 1:** Clinical and demographic variables in total sample and in the two MS subgroups.

Variables	Total sample (*N* = 63)	Range	ITG group (*N* = 32)	MTG group (*N* = 31)	*U*	*p*-Value^∗^
Age (years)^b^	48.27 ± 10.08 (49)	23–65	50.22 ± 8.69 (51.50)	46.20 ± 11.31 (58.50)	402.500	0.198
Gender (F/M, n)^c^	38/25	–	20/12	18/13	–	–
Education (years)^b^	12.24 ± 4.11 (13)	5–18	13.22 ± 3.44 (13)	11.17 ± 4.60 (9)	363.500	0.058
Disease duration (months)^b^	209.39 ± 117.09 (174)	24.0–504.0	212.57 ± 105.25 225)	205.99 ± 130.28 (167)	442.000	0.592
EDSS^a^	5.48 ± 1.47 (6)	1.5–7.5	5.76 ± 1.17 (6)	5.16 ± 1.71 (5.75)	416.500	0.268
Relapsing remitting (n)	9	–	4	5	–	–
Progressive form (n)	54	–	28	26	–	–

*^a^EDSS, Expanded Disability Status Scale. ^b^Values are mean ± SD; the numbers in brackets represent the median values; Values are frequency^c^ (Patient’s group were not significantly different for gender, Pearson x^2^ test = 0.129; p = 0.459); **^∗^**p-value, intergroup difference = Mann-Whitney U-test.*

In the whole sample the mean number of impaired neuropschological tests (6.06 ± 4.02) demonstrated a generally moderate cognitive impairment, without significant differences between the two groups (ITG group = 5.88 ± 4.17 and MTG group = 6.27 ± 3.90; Mann–Whitney *U*-test = 446.55, *p* = 0.636).

The mean raw scores of neuropsychological tests at baseline did not differ significantly between groups, except for SRT of Rao’s Battery, which was marginally significantly lower in MTG group compared to ITG group (**Supplementary Tables 1**, **2**). At the baseline the two groups did not differ for motor performance and psychological/emotional features (**Supplementary Tables 3**, **4**).

#### Group Comparison at Follow-Up

After Rehabilitation, the mean number of impaired neuropsychological tests was significantly lower in ITG group (ITG group = 3.97 ± 3.65, MTG group = 7.40 ± 3.97; Mann–Whitney *U*-test = 241.00, *p* = 0.001).

After intervention (T1), the ITG group showed significant improvements on several neuropsychological tests compared to baseline performance (**Tables 2**, **3**), whereas the MTG group significantly improved on two neuropsychological tests only (Rao Battery: Word List Generation, *p* = 0.030 and Selective Reminding Test-Delayed, *p* = 0.031; **Table 2**).

**Table 2:** Comparisons of raw scores of Italian Version Rao’s Brief Repeatable Battery at baseline (Pre-Test) and after rehabilitation (Post-Test) within each group.

Variables	ITG group (*N* = 32)			MTG group (*N* = 31)		
	Mean ± SD	Median	*p*-Value^∗^	Mean ± SD	Median	*p*-Value^∗^
SRT-LTS (pre)	23.69 ± 13.93	22.00	**0.009**	18.33 ± 11.61	17.50	0.124
SRT-LTS (post)	31.58 ± 14.50	32.00		22.00 ± 12.22	22.00	
SRT-CLTR (pre)	15.81 ± 11.96	15.00	**0.007**	10.90 ± 10.19	8.50	0.837
SRT-CLTR (post)	23.00 ± 12.59	22.00		12.92 ± 12.31	10.00	
SRT-D (pre)	5.41 ± 2.27	5.00	**0.001**	4.20 ± 2.51	4.00	**0.031**
SRT-D (post)	7.81 ± 4.57	8.00		5.40 ± 2.17	5.00	
SPART (pre)	15.19 ± 4.66	15.00	**0.001**	15.77 ± 4.73	15.00	0.638
SPART (post)	19.72 ± 5.84	21.00		17.28 ± 5.05	18.00	
SPART-D (pre)	5.06 ± 2.50	5.00	**0.001**	5.37 ± 2.09	6.00	1.000
SPART-D (post)	7.48 ± 4.66	7.00		5.36 ± 2.23	6.00	
WLG (pre)	18.19 ± 5.60	17.00	**0.002**	15.53 ± 5.68	15.00	**0.030**
WLG (post)	22.65 ± 7.33	23.00		18.44 ± 5.28	18.00	
SDMT (pre)	31.69 ± 10.65	32.00	**0.018**	30.27 ± 11.62	28.00	0.754
SDMT (post)	35.03 ± 11.22	35.00		31.00 ± 10.44	31.00	
PASAT 3″ (pre)	27.06 ± 12.64	26.00	**0.001**	24.43 ± 14.72	27.50	0.939
PASAT 3″ (post)	33.29 ± 12.80	34.00		26.36 ± 13.46	27.00	
PASAT 2″ (pre)	18.50 ± 10.53	20.00	**0.024**	19.60 ± 11.98	22.00	0.979
PASAT 2″ (post)	21.74 ± 11.83	23.00		20.36 ± 10.43	21.00	

*SRT-LTS, Selective Reminding Test-Long Term Storage; SRT- CLTR, Selective Reminding Test-Consistent Long Term Retrieval; SRT-D, Selective Reminding Test-Delayed; SPART, Spatial Recall Test; SPART-D, Spatial Recall Test-Delayed; WLG, Word List Generation; SDMT, Symbol Digit Modalities Test; PASAT-3, Paced Auditory Serial Addition Test-3 seconds; PASAT-2, Paced Auditory Serial Addition Test-2 seconds. Values are mean ± SD and Median. ^∗^p < 0.05 for intra-group difference. Intragroup differences were analyzed by Wilconxon sign rank test to determine the improvement after training. Statistically significant p-values are in bold.*

**Table 3:** Comparisons of raw scores the other neuropscyhological tests at baseline (Pre-Test) and after rehabilitation (Post-Test) within each group.

Variables	ITG group (*N* = 32)			MTG group (*N* = 31)		
	Mean ± SD	Median	*p*-Value^∗^	Mean ± SD	Median	*p*-Value^∗^
Spatial span (pre)	4.10 ± 0.59	4.00	**0.003**	4.36 ± 0.951	4.00	0.439
Spatial span (post)	4.55 ± 0.624	4.00		4.24 ± 0.723	4.00	
Forward verbal span (pre)	4.97 ± 0.795	5.00	**0.032**	5.07 ± 1.08	5.00	0.384
Forward verbal span (post)	5.35 ± 0.755	5.00		4.88 ± 0.927	5.00	
Backward verbal span (pre)	3.74 ± 0.96	3.00	**0.027**	3.86 ± 1.23	3.50	0.380
Backward verbal span (post)	4.13 ± 0.991	4.00		3.68 ± 1.14	3.00	
Stroop test: time (pre)	24.41 ± 13.61	20.50	0.607	27.57 ± 22.00	21.50	0.163
Stroop test: time (post)	22.75 ± 14.19	19.50		34.56 ± 28.17	28.50	
Stroop test: error (pre)	0.758 ± 2.20	0.000	0.438	1.66 ± 4.85	0.000	0.488
Stroop test: error (post)	0.35 ± 1.19	0.000		0.96 ± 1.48	0.000	
Phonological fluency (pre)	27.32 ± 12.10	28.00	**<0.001**	26.96 ± 12.54	25.50	0.134
Phonological fluency (post)	35.87 ± 12.89	37.00		28.92 ± 9.36	25.00	
FAB (pre)	14.97 ± 3.09	16.00	**0.009**	13.68 ± 3.17	14.00	0.921
FAB (post)	15.90 ± 2.56	17.00		14.60 ± 2.30	15.00	
Raven’s matrices (pre)	27.71 ± 5.12	29.00	**0.110**	25.93 ± 6.69	25.50	0.383
Raven’s matrices (post)	29.23 ± 3.94	30.00		26.72 ± 5.83	27.00	

*FAB, Frontal Assessment Battery. Values are mean ± SD and Median. ^∗^p < 0.05 for intra-group difference. Intragroup differences were analyzed by Wilconxon sign ranks test to determine the improvement after training. Statistically significant p-values are in bold.*

After intervention (T1), the ITG group showed significant improvements on both equilibrium (*p* < 0.001) and gait scores (*p* = 0.027) of the Tinetti scale, whereas performance of MTG group remained stable (**Table 4**). ITG groups significantly improved also on the BIM (ITG group: *p* = 0.016; **Table 4**).

**Table 4:** Comparisons of raw score of motor performance tasks at baseline (Pre-Test) and after rehabilitation (Post-Test) within each group.

Variables	ITG group (*N* = 32)			MTG group (*N* = 31)		
	Mean ± SD	Median	*p*-Value^∗^	Mean ± SD	Median	*p*-Value^∗^
Barthel Index Modified (Pre)	70.81 ± 16.96	79.00	**0.016**	72.16 ± 26.14	73.00	**0.020**
Barthel Index Modified (Post)	74.16 ± 16.67	80.00		71.68 ± 26.06	73.00	
Tinetti Balance Scale (Pre)	7.13 ± 3.60	7.00	**<0.001**	7.60 ± 3.60	8.00	0.763
Tinetti Balance Scale (Post)	8.77 ± 3.81	9.00		7.56 ± 3.57	7.00	
Tinetti Gait Scale (Pre)	5.74 ± 3.44	6.00	**0.027**	6.12 ± 3.5	6.00	0.157
Tinetti Gait Scale (Post)	6.58 ± 3.30	7.00		5.96 ± 3.44	6.00	
Tinetti Overall Scale (Pre)	12.71 ± 7.03	12.00	**<0.001**	13.72 ± 6.86	14.00	0.334
Tinetti Overall Scale (Post)	15.29 ± 6.49	15.00		13.52 ± 6.69	14.00	

*Values are mean ± SD and Median. ^∗^p < 0.05 for intra-group difference. Intragroup differences were analyzed by Wilconxon sign ranks test to determine the improvement after training. Statistically significant p-values are in bold.*

As for psychological features, State Anxiety decreased in both groups (ITG group: *p* = 0.006; MTG group: *p* = 0.005; **Table 5**). Moreover, the ITG group significantly improved on Fatigue Scale (*p* = 0.019) and BDI-II scale (*p* = 0.011), on both somatic-affective symptoms (*p* = 0.007) and cognitive symptoms (*p* = 0.031; **Table 5**).

**Table 5:** Comparisons of raw scorese of psychological scales at baseline (Pre-Test) and after rehabilitation (Post-Test) within each group.

Variables	ITG group (*N* = 32)			MTG group (*N* = 31)		
	Mean ± SD	Median	*p*-Value^∗^	Mean ± SD	Median	*p*-Value^∗^
FSS (pre)	4.96 ± 1.70	5.22	**0.019**	6.66 ± 10.78	4.50	0.762
FSS (post)	4.44 ± 1.39	4.66		4.45 ± 1.53	4.11	
BDI-II: total score (pre)	19.17 ± 12.48	18.00	**0.011**	18.40 ± 11.91	16.00	0.549
BDI-II: total score (post)	15.84 ± 12.17	15.00		18.80 ± 12.35	16.00	
BDI-II: cognitive (pre)	6.97 ± 5.77	6.00	**0.031**	6.68 ± 6.00	6.00	0.867
BDI-II: cognitive (post)	5.90 ± 5.71	5.00		6.36 ± 5.81	5.00	
BDI-II: somatic (pre)	12.21 ± 7.31	11.00	**0.007**	11.72 ± 6.49	11.00	0.343
BDI-II: somatic (post)	9.94 ± 7.26	9.00		12.40 ± 7.17	11.00	
STAI-Y: state (pre)	48.24 ± 12.56	48.00	**0.006**	47.20 ± 12.88	45.00	**0.005**
STAI-Y: state (post)	42.55 ± 11.68	41.00		40.24 ± 12.70	39.00	
STAI-Y: trait (pre)	47.93 ± 12.37	50.00	0.508	49.16 ± 14.02	48.00	0.493
STAI-Y: trait (post)	46.94 ± 11.76	48.00		47.16 ± 12.64	48.00	

*FSS, Fatigue Severity Scale; BDI-II, Beck Depression Inventory-II; STAI-Y, State-Trait Anxiety Inventory. Values are: mean ± SD and Median. ^∗^p < 0.05 for intra-group difference. Intragroup differences were analyzed by Wilconxon sign ranks test to determine the improvement after training. Statistically significant p-values are in bold.*

The advantage of ITG group was confirmed also by analyzing Delta (T1–T0) for the number of pathological tests which was significantly lower in ITG Group (−1.906 ± 2.531) compared to MTG group (1.133 ± 3.014; Mann–Whitney *U*-test = 199,500, *p* = < 0.001).

**FIGURE 2:**
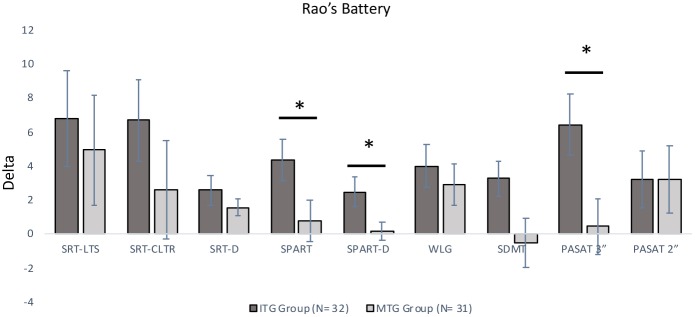
Comparisons of Delta (Post Test–Pre Test) scores for Italian Version Rao’s Brief Repeatable Battery (BRB) between the two groups (^∗^significantly different between groups; *p-corrected* for False Discovery Rate, Mann–Whitney *U*-test). Positive values indicate an improvement in performance; negative values indicate a worsening in performance. All bars mean standard error. SRT-LTS, Selective Reminding Test-Long Term Storage; SRT-CLTR, Selective Reminding Test-Consistent Long Term Retrieval; SRT-D, Selective Reminding Test-Delayed; SPART, Spatial Recall Test; SPART-D, Spatial Recall Test-Delayed; WLG, Word List Generation; SDMT, Symbol Digit Modalities Test; PASAT-3, Paced Auditory Serial Addition Test-3 seconds; PASAT-2, Paced Auditory Serial Addition Test-2 seconds.

**FIGURE 3:**
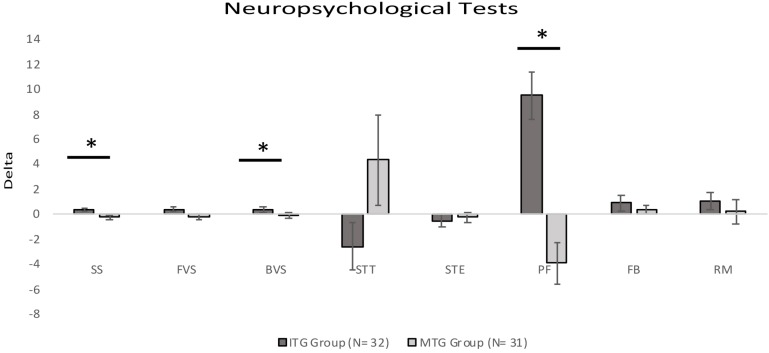
Comparisons of Delta (Post Test–Pre Test) scores for neuropsychological tests between the two groups (^∗^significantly different between groups; *p-corrected* for False Discovery Rate; Mann-Whitney *U*-test). Positive values indicate an improvement in performance; negative values indicate a worsening in performance. For the Stroop test time, positive values indicate a worsening in performance; negative values indicate an improvement in performance. All bars mean standard error. SS, Spatial Span; FVS, Forward Verbal Span; BVS, Backward Verbal Span; STT, Stroop Test Time; STE, Stroop Test Error; PF, Phonological fluency; FAB, Frontal Assessment Battery; RM, Raven’s Matrices.

**FIGURE 4:**
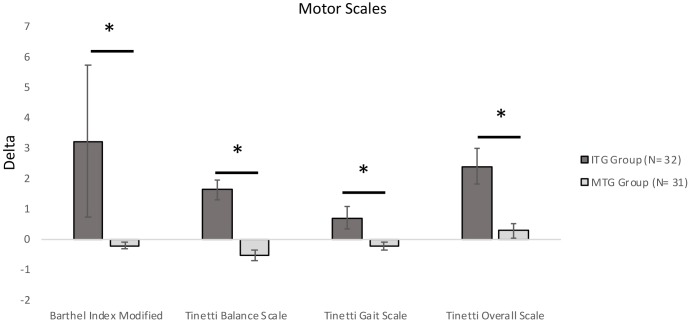
Comparisons of Delta (Post Test–Pre Test) scores for motor scales between the two groups (^∗^significantly different between groups; *p-corrected* for False Discovery Rate; Mann–Whitney *U-test*). Positive values indicate an improvement in performance; negative values indicate a worsening in performance. All bars mean standard error.

**FIGURE 5:**
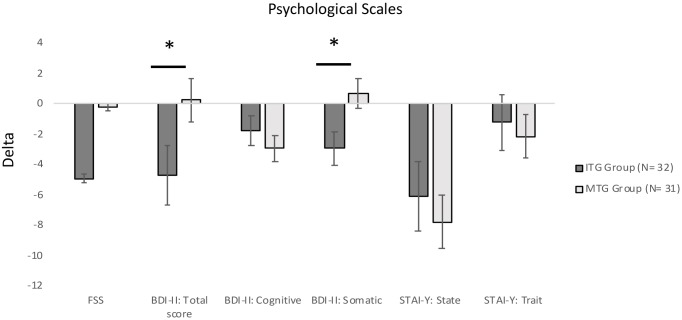
Comparisons of Delta (Post Test–Pre Test) scores for psychological scale between the two groups (^∗^significantly different between groups; ^∗^*p-corrected* for False Discovery Rate; Mann–Whitney *U-test)*. Positive values indicate increased scores; negative values indicate reduced scores. All bars mean standard error. FSS, Fatigue Severity Scale; BDI-II, Beck Depression Inventory-II; STAI-Y, State-Trait Anxiety Inventory.

ITG and MTG groups significantly differed on Delta scores on three neuropsychological tests included in the Rao Battery (**Figure 2**): Spatial Recall (SPART: Mann–Whitney *U*-test = 279.00, *corrected-p-value* = 0.027), and Spatial Recall and Delayed Test (SPART-D: Mann–Whitney *U*-test = 307.0*, corrected-p-value* = 0.027), assessing visuospatial learning and long term recall; PASAT Test 3″ (Mann–Whitney *U*-test = 305.00, *corrected-p-value* = 0.027), tapping divided attention and information processing speed. Moreover, ITG and MTG groups differed for the changes in the following three neuropsychological test scores (**Figure 3**): Spatial short-term memory span (Mann–Whitney *U*-test = 330.0, corrected*-p-value* = 0.036) and Backward verbal span (Mann–Whitney *U*-test = 304.5*,corrected-p-value* = 0.037), assessing divided attention and working memory; phonological fluency (Mann–Whitney *U*-test = 275.50, *corrected-p-value* = 0.016) assessing cognitive flexibility. We also observed significantly different Delta scores on Tinetti Overall Scale score (Mann–Whitney *U*-test = 141.50, *corrected-p-value* = 0.001), on equilibrium (Mann–Whitney *U*-test = 174.00, *corrected-p-value* = 0.001) and gait scores of Tinetti scale (Mann–Whitney *U*-test = 252.50, *corrected-p-values* = 0.001), and on independence in the activities of daily life (BIM; Mann–Whitney *U*-test = 178.50*, p* < 0.001; **Figure 4**). In reference to psychological scales (**Figure 5**), Delta for BDI-II scale was significantly different (Mann–Whitney *U*-test = 323.50, *corrected-p-values* = 0.039) in the two groups, particularly for somatic-affective symptoms (Mann–Whitney *U*-test = 271.500, *corrected-p-values* = 0.039).

The between-group differences related to training were confirmed by multiple linear regression analysis on the Delta for the number of pathological neuropsychological tests, taking into account the following confounding variables: group (Factor), EDSS, age, Delta for depression (BDI scale), Delta for fatigue (FSS scale) and Delta for motor functioning (Tinetti scale). Only type of treatment (β = −0.361, *t* = −2.520, *p* = 0.015) and age (β = −0.304, *t* = −2.389, *p* = 0.021) were significantly associated with the changes in the number of impaired tests.

The linear regression analysis performed to determine predictors of motor change (score of Tinetti Scale), taking into account group (Factor), EDSS, age, as well as Delta for BDI, FSS and number of pathological cognitive tests, showed that only type of treatment was significantly associated with the change in motor scale (β = 0.386; *t* = 2.541, *p* = 0.014).

## Discussion

The purpose of this prospective, multicenter, observational study was to examine the efficacy of the ICNR versus a traditional neuromotor training in a sample of MS patients. Our findings suggest that an “integrated rehabilitative approach” could improve both motor and cognitive skills, and reduce the somatic component of depressive symptoms. The present findings appear to be relevant because most patients were affected by progressive MS (54 of total sample: 28 of ITG Group and 26 of MTG Group). Indeed, several studies demonstrated effectiveness of cognitive rehabilitation in relapsing-remitting MS, whereas only a few data are available on cognitive rehabilitation in progressive MS (Briken et al., 2014; D’Amico et al., 2016).

The high number of patients affected by progressive MS in our sample might also account for the lack of substantial improvements in the group undergoing neuromotor therapy only. Indeed, the beneficial effect of traditional neuromotor rehabilitation in people with MS is well-established (Campbell et al., 2016), but it is less compelling when the level of disability increases (Hogan and Coote, 2009). Nonetheless, even in the group undergoing neuromotor therapy only we did not observe significant worsening of motor performance, and this could be considered as a relatively good outcome in patients with progressive MS. These findings are in line with previously published reviews showing that multi-disciplinary rehabilitation programs increased participation (as a result of a decrease in disability) and quality of life in people with MS (Khan et al., 2007).

We found that a multi-domain computer-assisted cognitive program supported by paper–pencil tasks significantly reduced the number of impaired neuropsychological tests and in general improved neuropsychological scores (Mattioli et al., 2015). Instead, we did not observe relevant cognitive improvements in the motor group (MTG).

Marked improvements in the ITG group were seen on tests tapping processing speed, attention, and cognitive flexibility (*PASAT-3, Backward Verbal Span and Phonological Fluency)*. Improvements in attention were consistent with previous studies on sustained attention (Amato et al., 2014) and on attention and executive functions (Mattioli et al., 2012) after cognitive rehabilitation. Notwithstanding some conflicting data about the effect of cognitive rehabilitation on executive functioning in MS, the studies implementing computerized cognitive rehabilitation programs often reported significant improvements in attention, information processing speed, working memory and executive functioning domains (Rilo et al., 2016; Pérez-Martín et al., 2017). Differences across studies may be explained by different conceptualisation, training, and assessment methods. We used a combined cognitive training mainly based on an Italian computerized training method different from that used in previous trials, but we could confirm that this kind of exercises can improve executive functioning and particularly verbal fluency, that was included in the present rehabilitative program.

Patients in the ITG group showed significantly larger improvements in both short- and long-term spatial memory compared to the control group. Spatial working memory has been relatively neglected in MS research and some studies suggested that improvement in attentional and executive functions had a role in improving performance on spatial memory tests (Gmeindl and Courtney, 2012). However, it is also possible that cognitive stimulation by spatial task may improve dexterity and movement coordination, increase strength, and help developing adaptive body movements during ambulation, transfers, self-care, and other functional activities as reported in patients with stroke (Barrett and Muzaffar, 2014). It is not surprising that spatial stimulation affects motor systems; visual-motor, integrative brain activity stimulates reorganization, so we suggest that spatial retraining as part of motor therapy could improve efficacy of in-hospital MS rehabilitation (Barrett and Muzaffar, 2014). However, the present preliminary association between motor recovery and spatial memory in MS should be verified in further studies, in which inclusion of a control group performing cognitive training only would allow to explore directionality of cross-modal trasfer (motor versus cognitive and cognitive versus motor).

Our results confirmed the hypothesis (Prosperini et al., 2015b) according to which the use of complex task-specific training may promote re-learning capacity, possibly inducing functional or structural plasticity in brain networks controlling both motor and cognitive functions. The so-called ‘far transfer effect’ or ‘transfer of training’ refers to the occurrence of transferring improvements in a specific function to untrained functional domains. In the present study the ITG group received cognitive stimulation and motor rehabilitation and improved significantly in executive and attentional functions and in some motor functions (gait and balance) compared to the group that received neuromotor therapy only. Such findings would be consistent with the idea of a cognitive versus motor cross-modal transfer.

Deficits in attention and executive function processes have been independently associated not only with deteriorated walking performance, but also with postural instability and future falls in MS (Kalron, 2014) suggesting that motor skills, balance and cognition share common resources. Postural control and cognition compete for a common pool of attentional resources and when one task is made more challenging, or when neural networks become less efficient, available resources reach their limit (Leone et al., 2015). For this reason, cognitive rehabilitation and neuromotor training might have had positive effects on cognitive and motor outcomes compared with single modes of rehabilitation (Briken et al., 2014). In other terms, 1 h of combined cognitive and motor treatment seemed to be more effective than 1 h of motor treatment alone, thus suggesting that combined rehabilitative approaches not only determine more favorable cognitive and motor outcomes but might be considered as the best choice in terms of time and costs for health policy planning.

Nonetheless, many unanswered questions remain about combined interventions in MS, and about efficacy of exercise training and cognitive rehabilitation interventions on walking and cognition as a function of clinical characteristics, such as disability status, or domain of cognitive impairment (Motl et al., 2016).

The neurobehavioral sequelae of MS consistently include fatigue, clinical depression, and cognitive dysfunction (Diamond et al., 2008). In the present study we observed moderate levels of depression in both groups, but depressive symptomatology, and particularly somatic symptoms, improved only in the ITG group. This finding underlines the link between depression and cognitive performance in MS (Portaccio, 2016). At the behavioral level, depression seems to alter attentional capacity, working memory and executive functions. This interaction is not surprising, considering the overlap between emotional regulatory regions and the executive network, and the results of neuroimaging studies in depressed patients with MS reporting an involvement of frontal areas (Portaccio, 2016). However, a dysexecutive syndrome secondary to MS might precipitate depression, by altering emotional processing and favoring use of maladaptive cognitive strategies. Indeed, there is a complex interplay between cognition, depression and fatigue in MS, with each symptom impacting negatively on the others. Our results suggested that integrated cognitive and motor rehabilitation can exert a positive effect on depression in MS, as in patients with traumatic brain injury (Kumar et al., 2018). Indeed, regression analyses suggested that the improvements in cognitive performance were independent from changes in depression and fatigue. Only age was inversely correlated with improvements in the number of pathological tests, in agreement with studies showing that age reduces brain plasticity in MS patients (Zeydan and Kantarci, 2018).

Cognitive, emotional and motor processes depend on a series of integrated and highly interconnected brain circuits. The mechanisms through which cognitive and motor rehabilitation could improve cognition in MS are not well-understood, although it has been proposed that specific interventions might stimulate neural pathways through neuroplasticity (Penner et al., 2007). On the basis of the “mind/brain full correspondence principle” (Grossi et al., 2017) it is possible to act simultaneously on multiple dimensions through psychotherapy, cognitive training, pharmacology and neurostimulation techniques, in order to modulate activity of altered networks. Neurofunctional studies recently demonstrated that rapid-onset plasticity and functionally relevant chronic reorganization processes are preserved even in progressive form of MS and that these phenomena are functionally relevant to preserve motor and cognitive functions (Schoonheim et al., 2015).

In summary, this study provided the first evidence for beneficial effects of “integrated training” on walking ability, cognitive function and neuropsychiatric symptoms in patients with progressive MS and moderate-to-advanced disability. Given the limited pharmacological treatment options for progressive MS, further investigation of interventions in this clinical form is clearly warranted. Although intriguing, our findings have to be interpreted cautiously for several reasons: (1) the relatively small sample size limits generalizability of the results and reduces the observed power; (2) as logistic factors determined patients’ assignment, the lack of a randomized assignment to one treatment group might have introduced unwanted sampling biases. This second limitation was tempered by the fact that the two groups did not differ for their main demographic, clinical, cognitive, and psychological characteristics.

Further studies including larger and more representative samples of MS patients are needed to confirm the present promising results which suggest that combined rehabilitation approaches addressing both cognitive and motor systems are efficient, address areas of great need, and could improve intensive treatment in MS rehabilitation. Long-term follow-up studies are necessary to ascertain long-term stability of cognitive improvements and their generalization to everyday functional activities.

## Author Contributions

All authors listed have made substantial, direct, and intellectual contribution to the work, and approved its final version for publication. AMB conceived and designed the study, analyzed and interpreted the data, drafted and critically reviewed the manuscript, and performed as coordinator of neuropsychological aspects of the work. GL conceived and designed the study, supervised the study, recruited the patients, and critically reviewed the manuscript. ES analyzed and interpreted the data, recruited the patients, and critically reviewed the manuscript. LT conceived and designed the study, analyzed and interpreted the data, and critically reviewed the manuscript. DG conceived and designed the study, supervised the study, critically revised the manuscript, and performed as theoretical coordinator of rational basis. ME evaluated the patients and revised the manuscript. TC evaluated and selected the patients, and revised the manuscript. RL and FS interpreted the data, recruited the patients, and revised the manuscript. VBM conceived and designed the study, supervised the study, recruited the patients, and critically revised the manuscript. GC conceived and designed the study, critically reviewed the manuscript, and performed as coordinator of rehabilitation aspects of the work.

## Conflict of Interest Statement

The authors declare that the research was conducted in the absence of any commercial or financial relationships that could be construed as a potential conflict of interest.
