# Supplementary material for: Integrated Cognitive and Neuromotor Rehabilitation in Multiple Sclerosis: A Pragmatic Study
Source: Front Behav Neurosci. 2018 Sep 5;12:196. doi: 10.3389/fnbeh.2018.00196 (PMC6146227; doi:10.3389/fnbeh.2018.00196)
Supplement: Supplementary file 1 [file Table_1.DOCX]

**Table 1. Scores on the Italian Version Rao’s Brief Repeatable Battery in the two subgroups at baseline**

| **Variables** | **ITG Group (N= 32)** | | **MTG Group (N= 31)** | |  | |
| --- | --- | --- | --- | --- | --- | --- |
|  | mean ± SD | Median | mean ± SD | Median | U | p-Value* |
| SRT-LTS | 23.69 ±13.93 | 22.00 | 18.33 ±11.61 | 17.50 | 401.50 | .193 |
| SRT-CLTR | 15.81 ±11.96 | 15.00 | 10.90 ±10.19 | 8.50 | 379.00 | .107 |
| SRT-D | 5.41 ±2.27 | 5.00 | 4.20 ±2.51 | 4.00 | 340.00 | .030 |
| SPART | 15.19 ±4.66 | 15.00 | 15.77 ±4.73 | 15.00 | 474.50 | .767 |
| SPART-D | 5.06 ±2.50 | 5.00 | 5.37 ±2.09 | 6.00 | 464.00 | .657 |
| WLG | 18.19 ±5.60 | 17.00 | 15.53 ±5.68 | 15.00 | 368.00 | .078 |
| SDMT | 31.69 ±10.65 | 32.00 | 30.27 ±11.62 | 28.00 | 444.00 | . 612 |
| PASAT 3” | 27.06 ±12.64 | 26.00 | 24.43 ±14.72 | 27.50 | 455.00 | .572 |
| PASAT 2” | 18.50 ±10.53 | 20.00 | 19.60 ±11.98 | 22.00 | 458.00 | .600 |

**Note:** **SRT-LTS**, Selective Reminding Test-Long Term Storage; **SRT- CLTR**, Selective Reminding Test-Consistent Long Term Retrieval; **SRT-D**, Selective Reminding Test-Delayed; **SPART**, Spatial Recall Test; **SPART-D**, Spatial Recall Test-Delayed; **WLG**, Word List Generation; **SDMT**, Symbol Digit Modalities Test; **PASAT-3**, Paced Auditory Serial Addition Test-3 seconds; **PASAT-2**, Paced Auditory Serial Addition Test-2 seconds.

The two groups differed at baseline only on SRT-D; all other p>.05) **^**^**p value, intergroup difference = U-*Mann-Whitney test*
