# Supplementary material for: Integrated Cognitive and Neuromotor Rehabilitation in Multiple Sclerosis: A Pragmatic Study
Source: Front Behav Neurosci. 2018 Sep 5;12:196. doi: 10.3389/fnbeh.2018.00196 (PMC6146227; doi:10.3389/fnbeh.2018.00196)
Supplement: Supplementary file 2 [file Table_2.DOCX]

**Table 2. Scores on the other neuropscyhological tests in the two subgroups at baseline**

| **Variables** | **ITG Group (N= 32)** | | | **MTG Group (N= 31)** | | |  | | |  |
| --- | --- | --- | --- | --- | --- | --- | --- | --- | --- | --- |
|  | | mean ± SD | Median | | mean ± SD | Median | | U | p-Value* | |
| Spatial Span | | 4.10 ±.59 | 4.00 | | 4.36 ±.951 | 4.00 | | 438.50 | .354 | |
| Forward Verbal Span | | 4.97 ±.795 | 5.00 | | 5.07 ±1.08 | 5.00 | | 480.00 | . 817 | |
| Backward Verbal Span | | 3.74 ±.96 | 3.00 | | 3.86 ±1.23 | 3.50 | | 454.00 | .865 | |
| Stroop Test: Time | | 24.41 ±13.61 | 20.50 | | 27.57 ±22.00 | 21.50 | | 468.00 | .866 | |
| Stroop Test: Error | | .758 ±2.20 | .000 | | 1.66 ±4.85 | .000 | | 377.00 | .070 | |
| Phonological fluency | | 27.32 ±12.10 | 28.00 | | 26.96 ±12.54 | 25.50 | | 457.50 | .596 | |
| FAB | | 14.97 ±3.09 | 16.00 | | 13.68 ±3.17 | 14.00 | | 373.00 | .087 | |
| Raven’s Matrices | | 27.71 ±5.12 | 29.00 | | 25.93 ±6.69 | 25.50 | | 396.00 | .235 | |

**Note:** **FAB:** Frontal Assessment Battery. *p-value, intergroup difference = U-Mann-Whitney test
