# Supplementary material for: Integrated Cognitive and Neuromotor Rehabilitation in Multiple Sclerosis: A Pragmatic Study
Source: Front Behav Neurosci. 2018 Sep 5;12:196. doi: 10.3389/fnbeh.2018.00196 (PMC6146227; doi:10.3389/fnbeh.2018.00196)
Supplement: Supplementary file 3 [file Table_3.DOCX]

**Table 3. Scores on motor performance tasks in the two subgroups at baseline**

| Variables | ITG Group (N= 32) | | MTG Group (N= 31) | |  | |
| --- | --- | --- | --- | --- | --- | --- |
|  | mean ± SD | Median | mean ± SD | Median | U | p-Value**^*^** |
| Barthel Index Modified | 70.81 ±16.96 | 79.00 | 72.16 ±26.14 | 73.00 | 331.00 | .267 |
| Tinetti Balance Scale | 7.13 ±3.81 | 7.00 | 7.60 ±3.60 | 8.00 | 348.50 | .405 |
| Tinetti Gait Scale | 5.74 ±3.44 | 6.00 | 6.12 ±3.5 | 6.00 | 369.50 | .622 |
| Tinetti Overall Scale | 12.71 ±7.03 | 12.00 | 13.72 ±6.86 | 14.00 | 346.00 | .493 |

**Note.** The two groups did not differ at baseline for any measure (all p>.05). **^*^**p value, intergroup difference = U-*Mann-Whitney test*
