# Supplementary material for: Integrated Cognitive and Neuromotor Rehabilitation in Multiple Sclerosis: A Pragmatic Study
Source: Front Behav Neurosci. 2018 Sep 5;12:196. doi: 10.3389/fnbeh.2018.00196 (PMC6146227; doi:10.3389/fnbeh.2018.00196)
Supplement: Supplementary file 4 [file Table_4.DOCX]

**Table 4. Scores on Psychological Scales in the two subgroups at baseline**

| **Variables** | **ITG Group (N= 32)** | | **MTG Group (N= 31)** | |  | |
| --- | --- | --- | --- | --- | --- | --- |
|  | mean ± SD | Median | mean ± SD | Median | U | p-Value**^*^** |
| FSS | 4.96 ±1.70 | 5.22 | 6.66 ±10.78 | 4.50 | 375.00 | .270 |
| BDI-II: Total score | 19.17 ±12.48 | 18.00 | 18.40 ±11.91 | 16.00 | 446.50 | .800 |
| BDI-II: Cognitive | 6.97 ±5.77 | 6.00 | 6.68 ±6.00 | 6.00 | 433.50 | .659 |
| BDI-II: Somatic | 12.21 ±7.31 | 11.00 | 11.72 ±6.49 | 11.00 | 435.00 | .675 |
| STAI-Y: State | 48.24 ±12.56 | 48.00 | 47.20 ±12.88 | 45.00 | 356.00 | .910 |
| STAI-Y: Trait | 47.93 ±12.37 | 50.00 | 49.16 ±14.02 | 48.00 | 356.00 | .910 |

**Note**. **FSS**: Fatigue Severity Scale; **BDI-II**: Beck Depression Inventory; **STAI-Y**: State-Trait Anxiety Inventory.

The two groups did not differ at baseline for any measure (all p>.05) **^*^**p value, intergroup difference = U-*Mann-Whitney test*
